# Identification of *RPA3* as a Potential Functional Effector of Chromosome 7 Gain in Glioblastoma

**DOI:** 10.3390/biomedicines14051014

**Published:** 2026-04-30

**Authors:** Yulu Ge, Zhan Hu, Wenbo Wu, Wenbin Ma, Tingyu Liang, Yu Wang

**Affiliations:** 1 Department of Neurosurgery, Center for Malignant Brain Tumors, National Glioma MDT Alliance, Peking Union Medical College Hospital, Chinese Academy of Medical Sciences and Peking Union Medical College, Beijing 100730, China; ge-yl19@mails.tsinghua.edu.cn (Y.G.); wenbo_wu@student.pumc.edu.cn (W.W.); 2 Eight-Year Medical Doctor Program, Chinese Academy of Medical Sciences and Peking Union Medical College, Beijing 100730, China; hu-z20@mails.tsinghua.edu.cn

**Keywords:** glioblastoma, chromosome 7 gain, *RPA3*, DNA repair

## Abstract

**Background:** Chromosome 7 gain (chr7 gain) is a highly prevalent early event in glioblastoma (GBM). Because chr7 gain usually involves broad chromosomal amplification, its biological impact is unlikely to be fully explained by canonical loci such as *EGFR* and *MET*. The contribution of less-characterized, dosage-sensitive genes on chromosome 7 remains insufficiently defined. This study aimed to identify additional chr7 candidates associated with malignant phenotypes in GBM. **Methods:** Transcriptomic, copy-number, and clinical data from TCGA-GBM and TCGA-LGG were analyzed to characterize chr7-gain-associated alterations and prioritize candidate genes. Refined GBM and histologic GBM cohorts based on the WHO 2021 framework were used for candidate selection. *RPA3*-associated pathway features were examined using ssGSEA, PROGENy, WGCNA, and protein–protein interaction analysis, with external validation in the CGGA-693 cohort. Single-cell RNA-seq analysis compared chr7-gain and chr7-normal-copy tumor subclusters. Functional relevance was evaluated by siRNA-mediated knockdown in U87 and U118 cells. **Results:** Chr7 gain was enriched in high-grade IDH-wildtype gliomas and was associated with cell-cycle- and DNA repair-related programs. *RPA3* was prioritized as a dosage-sensitive chromosome 7 candidate based on its upregulation in chr7-gain tumors, association with poor prognosis, and concordance with replication- and repair-related signatures. In vitro, *RPA3* knockdown impaired cell growth, proliferation, colony formation, and migration. Single-cell analysis suggested greater transcriptomic and network-level relevance of *RPA3* in chr7-gain tumor cells. **Conclusions:** *RPA3* is a dosage-sensitive chromosome 7 candidate associated with aggressive and replication-/repair-related phenotypes in GBM. Increased *RPA3* expression may contribute to the selective advantage associated with chr7 gain, which supports further investigation as potential therapeutic target.

## 1. Introduction

Glioma is the most prevalent malignant central nervous system (CNS) tumor in adults, and it is characterized by diffuse infiltration into the surrounding brain parenchyma, marked cellular plasticity and inter- and intratumoral heterogeneity, and high resistance to therapy, which collectively contribute to rapid progression and high recurrence rates [1,2]. The highest grade of glioma, glioblastoma (GBM), with an incidence of 3.26 per 100,000 population, exhibits a median overall survival (OS) of less than 15 months despite standard treatment consisting of maximal safe resection followed by radiotherapy with concomitant and adjuvant temozolomide, reflecting its dismal prognosis [3,4]. Historically, the WHO 2016 classification established diagnoses and grading primarily through traditional histomorphological features. Mitotic activity and anaplastic nuclear characteristics distinguished WHO grade III from grade II gliomas, while the addition of microvascular proliferation and/or necrosis defined WHO grade IV (GBM) [5]. The updated 2021 WHO classification, however, redefines GBM diagnostically as an IDH-wildtype (IDH-wt) glioma exhibiting either conventional histological features or at least one of three molecular alterations (*TERT* promoter mutation, *EGFR* gene amplification, combined gain of chromosome 7 and loss of entire chromosome 10) [6]. This shift further emphasizes the role of molecular alterations in malignant phenotypes, and highlights the need for deeper investigation into molecular pathogenesis.

Gain of chromosome 7 (chr7 gain) and loss of chromosome 10 (chr10 loss) were copy-number alterations (CNAs) predominantly observed in IDH-wt GBM, detected in approximately 59% of cases [7]. However, despite its high prevalence and early occurrence during gliomagenesis, why chromosome 7 gain is selected and how it functionally contributes to malignant progression remain incompletely understood. Evolutionary studies have placed chr7 gain and chr10 loss among the earliest events in glioblastoma development [8,9], yet an important unresolved question is why tumor evolution repeatedly selects broad gain of the entire chr7 rather than only focal amplification of canonical drivers such as *EGFR* [8]. *EGFR* amplification was not consistently present in all GBMs with chr7 gain [9]. Gain of entire chromosome 7 or moderate polysomy showed no clear association with *EGFR* expression levels [10]. Recent research found that features that contribute the most to the recurrence patterns of aneuploidy observed in human cancer were the densities of oncogenes and tumor suppressor genes [11]. This indicates that there might exist a dense distribution of key oncogenes on chr7. To date, the chr7 loci most intensively investigated in glioma include canonical drivers such as *EGFR*, *MET*, and *PDGFA*, together with the *HOXA* region, which have been implicated in chr7 gain and aggressive tumor behavior [7,12]. However, these representative loci occupy only a small fraction of chr7, and the functional landscape of broad chr7 gain warrants investigation of additional dosage-sensitive genes.

Among genes located on chr7, replication protein A3 (*RPA3*) is biologically notable because it encodes the 14-kDa subunit of the heterotrimeric replication protein A (RPA) complex, the major eukaryotic single-stranded DNA-binding complex required for DNA replication, recombination, and repair [13,14]. During replication stress and DNA damage, RPA-coated single-stranded DNA protects vulnerable DNA intermediates from nuclease attack and inappropriate secondary-structure formation, while also serving as a dynamic platform for the recruitment of checkpoint and repair factors, thereby supporting replication fork stability and genome integrity [15,16,17]. This function is particularly relevant to tumor biology because rapidly proliferating cancer cells experience persistent endogenous replication stress and rely heavily on DNA damage response pathways to sustain survival and continued cell division [18,19], and elevated *RPA3* expression has been associated with aggressive behavior, poor prognosis, or treatment resistance in several malignancies [20,21,22]. Moreover, a recent study in glioma reported that *RPA3* is highly expressed and promotes proliferation, migration, and invasion [23]. Based on these observations, we hypothesized that *RPA3* may act as a functionally relevant, dosage-sensitive effector of chr7 gain in GBM.

In this study, we leveraged the TCGA-GBM and TCGA-LGG cohorts to systematically investigate the transcriptional alterations and inferred pathway activities induced by chr7 gain in glioma cells. By integrating copy-number-associated expression changes of chr7 genes with prognostic associations, we sought to identify candidate dosage-sensitive effectors that may contribute to the malignant phenotype of chr7-gain GBM. Given the biological relevance to DNA replication and repair, we prioritized *RPA3* for further evaluation. We then examined its expression pattern and clinical relevance in glioma, explored its potential functional significance, and further assessed its chr7-gain-specific importance through single-cell transcriptomic analysis. Through this approach, we aimed to determine whether *RPA3* represents a functionally relevant mediator of the selective advantage conferred by chr7 gain in GBM.

## 2. Materials and Methods

### 2.1. Clinical Cohorts and Data Acquisition

The Cancer Genome Atlas (TCGA) is a public consortium resource that provides uniformly processed multi-omic data and curated clinical annotations for human cancers. For diffuse gliomas, TCGA contains two companion projects: TCGA-GBM (glioblastoma, WHO grade IV tumors) and TCGA-LGG (low-grade gliomas, primarily WHO grade II–III). Bulk RNA-seq, copy-number segments, and clinical metadata for TCGA-GBM and TCGA-LGG were downloaded from the NCI Genomic Data Commons via TCGAbiolinks (v2.28.3) in R (v4.3.1). Arm-level copy-number calls generated by GISTIC2.0 were obtained via TCGAbiolinks. In the GISTIC framework, +1 and +2 indicate low-level and high-level amplification, respectively. We defined chr7 gain as concomitant arm-level gain of both 7p and 7q (GISTIC calls +1 or +2 on each arm). This definition was chosen because gain involving both chromosome 7 arms is more consistent with whole-chromosome 7 gain, which is the biologically and diagnostically relevant alteration highlighted in IDH-wt glioma and in the WHO framework. For external validation, bulk transcriptomic and corresponding clinical data from the CGGA-693 cohort were obtained from the Chinese Glioma Genome Atlas (CGGA), and the IDH-wildtype GBM subset was used for validation analyses. In addition, a public single-cell RNA-sequencing dataset of human glioblastoma was obtained from the Gene Expression Omnibus (GEO) under accession number GSE182109. Raw expression matrices were downloaded.

For pan cancer and tumor-versus-normal comparisons, we used GEPIA2 [24], an interactive web server based on UCSC Xena-recomputed TCGA and GTEx RNA-seq data processed through a uniform pipeline to improve cross-dataset comparability and reduce computational batch effects, to obtain expression summaries and basic differential analyses using log2(TPM+1)-transformed expression values; where differential-expression settings were applied, the GEPIA2 default thresholds of |log2FC| = 1 and q-value = 0.01 were used. *RPA3* copy-number and expression data across cell lines were retrieved from DepMap [25] and correlated with 7p arm-level copy-number to validate dosage effects. As this study solely involved the analysis of publicly available data and did not include any animal experiments, ethics approval was not applicable.

### 2.2. Analytical Workflow and Candidate-Gene Prioritization

This study used a stepwise workflow to identify dosage-sensitive chromosome 7 candidates associated with chr7 gain in glioma. First, TCGA-GBM and TCGA-LGG data were analyzed to define the distribution of chr7 gain and its associated transcriptomic alterations. Second, to reduce confounding from histological and molecular heterogeneity, candidate discovery was performed in two WHO 2021-based cohorts: a refined GBM cohort and a histologic GBM cohort. Within each cohort, differential expression analysis was used to identify genes upregulated in chr7-gain tumors, followed by GSEA and univariable Cox regression to prioritize chromosome 7 genes associated with both chr7 gain and poor survival. Third, overlapping prognostic candidates from the two cohorts were compared, and *RPA3* was prioritized because it showed dosage-associated upregulation, adverse prognostic association, and the closest biological concordance with the recurrent replication- and DNA repair-related signatures identified in chr7-gain GBM. Finally, downstream integrative analyses, including ssGSEA, PROGENy, WGCNA, drug-response prediction, protein–protein interaction analysis, external validation in CGGA, single-cell analysis, and in vitro experiments, were performed to further characterize the potential functional relevance of *RPA3*.

### 2.3. Differential Gene Expression

Raw count data were processed using the DESeq2 (v1.40.2) package in R. Low-count genes were filtered to remove genes with minimal expression, as retaining them could reduce the power to detect differentially expressed genes. Library sizes were normalized between samples using the DESeq2 median-of-ratios method to account for differences in sequencing depth. Differential expression analysis was performed using the DESeq2 framework, which employs a generalized linear model based on the negative binomial distribution. For the specified contrasts, the Wald test was used to assess statistical significance. The resulting *p*-values were adjusted for multiple testing using the Benjamini–Hochberg false discovery rate (FDR) procedure.

### 2.4. Pathway and Gene Set Enrichment Analysis

Gene Set Enrichment Analysis (GSEA) identifies coordinated expression changes across pre-defined gene sets without applying arbitrary significance thresholds to individual genes [26]. GSEA was conducted using the Hallmark gene sets from the Molecular Signatures Database (MSigDB) [27], and using the clusterProfiler package (v4.8.3), with genes ranked by their expression fold changes. Functional annotation of differentially expressed genes was also explored through Gene Ontology (GO) enrichment analysis and Kyoto Encyclopedia of Genes and Genomes (KEGG) pathway analysis [28,29]. For pathway activity quantification at the sample level, single-sample GSEA (ssGSEA) and PROGENy were utilized to calculate enrichment scores for each sample. The ssGSEA algorithm was performed using the Hallmark gene sets, and using the GSVA package (v1.5) [30]. The PROGENy method infers the activity of 14 core oncogenic signaling pathways, and computes a pathway activity score based on a consensus set of downstream responsive genes [31]. It was performed using the progeny package (v1.22).

### 2.5. Survival Analysis

Prognostic associations were assessed by univariable Cox regression, yielding hazard ratios (HRs) with 95% confidence intervals. Significant genes were then subjected to Kaplan–Meier (KM) analysis. Patients were stratified into high- and low-expression groups by the median value, and survival differences were evaluated with the log-rank test (reported as log *p*). The median overall survival (mOS) for each group was calculated. Analyses were performed using the survival (v3.5.7) and survminer (v0.4.9) R packages.

### 2.6. Weighted Gene Co-Expression Network Analysis (WGCNA)

Weighted Gene Co-expression Network Analysis (WGCNA) was performed using the WGCNA package (v1.72.1) to identify gene co-expression modules [32]. Within a module, module membership (MM) was calculated as the correlation between a gene and the module eigengene, while gene significance (GS) was defined as the correlation between a gene and the trait of interest. Intramodular hub genes were selected using the criteria of MM > 0.8 and GS > 0.2.

### 2.7. Drug Response Prediction

Drug sensitivity for the GBM samples was predicted using the R package oncoPredict (v0.2) [33]. The drug response models were trained on the Genomics of Drug Sensitivity in Cancer (GDSC2) database, which contains dose–response data from a large panel of cancer cell lines [34]. The trained model (implemented in the calcPhenotype function) leverages gene expression profiles to predict drug response. This model was then applied to our GBM transcriptomic dataset to estimate the half-maximal inhibitory concentration (IC50) for each drug–sample pair.

### 2.8. Protein–Protein Interaction (PPI) Network Construction

To identify functional interactions among the differentially expressed genes, a protein–protein interaction (PPI) network was constructed using the STRING database (v12.0) [35]. The gene list was submitted to STRING to retrieve interactions from the full, comprehensive network, where edges represent both functional associations and physical binding. A minimum required interaction score of 0.4 (medium confidence) was set as the threshold.

### 2.9. Single-Cell RNA-Seq Processing and Copy-Number Inference

Public single-cell RNA-seq data from GSE182109 were analyzed using the Seurat package (v.4.3.0) in R. Cells with a mitochondrial gene proportion greater than 5% were excluded during quality control, and the remaining cells were normalized, scaled, and subjected to principal component analysis (PCA). Cell clustering was performed using a shared nearest-neighbor graph-based approach, and clusters were visualized by uniform manifold approximation and projection (UMAP). To infer large-scale copy-number variation patterns across malignant subclusters, inferCNV (v.1.18.1) was performed. Based on the inferred chromosome 7 copy-number profiles, tumor subclusters showing clear gain of chromosome 7 were classified as chr7-gain subclusters, whereas subclusters lacking evident chromosome 7 gain were classified as chr7-normal-copy subclusters.

### 2.10. In Silico RPA3 Knockout Analysis

An in silico knockout analysis was performed using the scTenifoldKnk (v.1.0.1) [36] framework. Separate count matrices were generated from the selected chr7-gain and chr7-normal-copy tumor cells. *RPA3* was specified as the perturbed gene, and virtual knockout was performed independently in each group. The resulting changes in the gene regulatory network after virtual *RPA3* knockout were recorded for downstream visualization and analysis.

### 2.11. Cell Lines and Culture

Human glioblastoma cell lines U87 and U118 were purchased from the Institute of Biochemistry and Cell Biology, Chinese Academy of Science. Cells were cultured in Dulbecco’s Modified Eagle Medium (DMEM; Gibco, Waltham, MA, USA) supplemented with 10% fetal bovine serum (FBS; Biosharp Life Sciences, Hefei, China) at 37 °C with 5% CO_2_.

### 2.12. siRNA Transfection and Knockdown Validation

To knock down *RPA3* expression, two non-overlapping siRNA sequences (si*RPA3*-#1: 5′-CGAUUGUAAAUGAGCUAUAUU-3′; si*RPA3*-#2: 5′-CCACCAUCUUGUGUACAUCCU-3′) and a negative control siRNA were transfected into cells using the riboFECT CP Transfection Kit (RIBOBIO, Cat# 166T), following the manufacturer’s protocol. Knockdown efficiency was validated by quantitative RT-PCR (qRT-PCR). Total RNA was extracted 48 h post-transfection and reverse transcribed into cDNA using the RevertAid First Strand cDNA Synthesis Kit (ThermoFisher, Waltham, MA, USA). PCR amplification was then performed using PowerUp™ SYBR™ Green Master Mix (ThermoFisher). The relative expression of *RPA3* was calculated using the 2^−ΔΔCt^ method, normalized to the housekeeping gene GAPDH. Knockdown efficiency was expressed as the percentage reduction in normalized *RPA3* expression relative to the negative control group.

### 2.13. Cell Counting Kit-8 (CCK-8), EdU, and Colony-Forming Assays

Cell viability was assessed using the Cell Counting Kit-8 Cell Counting Kit-8 (CCK-8) assay (Dojindo Laboratories, Kumamoto, Japan) according to the manufacturer’s protocol. Briefly, at each 24 h time point, the culture medium was replaced with 100 µL of fresh DMEM and 10 µL of CCK-8 solution, followed by incubation at 37 °C for 1 h. The absorbance at 450 nm was measured using a microplate reader. Cell proliferation was evaluated using the BeyoClick™ EdU Cell Proliferation Kit with Alexa Fluor 594 (Beyotime, Shanghai, China) according to the manufacturer’s instructions. The proportion of proliferating cells was determined by manual counting and calculated as the percentage of EdU-positive cells among all cells in each 10× fluorescence microscopy image. For the colony-formation assay, 2000 cells were seeded per well in 6-well plates and cultured for two weeks. The resulting colonies were fixed, stained with crystal violet, and manually counted.

### 2.14. Transwell and Wound Healing Assays

For transwell assay, cell migration was assessed using 12-well Transwell plates with 8 μm pore inserts (Corning Incorporated, Corning, NY, USA). Cells were resuspended in serum-free medium and seeded into the upper chamber. The lower chamber was filled with medium containing 20% FBS as a chemoattractant. After 24 h of incubation, non-migrated cells on the upper surface of the membrane were gently removed. The cells that had migrated to the lower surface were fixed, stained with 0.1% crystal violet, imaged, and quantified by ImageJ (v.1.54s)-based cell counting in five random fields per insert. For wound healing, a uniform wound was created in the monolayer by scratching with a 200 μL pipette tip. The detached cells and debris were washed away with PBS. The wells were then filled with fresh serum-free medium. Images of the wound area were captured at 0, 12, and 24 h using an inverted microscope.

### 2.15. Cell-Cycle Analysis by Flow Cytometry

Cell-cycle distribution was analyzed by propidium iodide (PI) staining followed by flow cytometry. Briefly, 48 h after siRNA transfection, U87 and U118 cells were harvested, washed twice with cold PBS, and fixed in 70% ethanol at 4 °C overnight. Fixed cells were then washed with PBS to remove residual ethanol and incubated with PI/RNase staining solution in the dark at room temperature for 30 min, according to the manufacturer’s instructions. DNA content was measured by flow cytometry, and the proportions of cells in G0/G1, S, and G2/M phases were calculated using standard cell-cycle modeling in FlowJo software (v10; FlowJo LLC, Ashland, OR, USA).

### 2.16. Statistical Analysis

A two-sided *p*-value < 0.05 was considered statistically significant. For all analyses involving multiple hypothesis testing, the Benjamini–Hochberg false discovery rate (FDR) procedure was applied, with an FDR < 0.05 deemed significant. Correlations were assessed using Pearson or Spearman methods as appropriate for the data distribution. For the cellular functional assays, data are presented as mean ± standard deviation (SD) from at least three independent biological replicates, each containing a minimum of three technical replicates unless otherwise specified. For two-group comparisons, unpaired two-tailed Student’s *t*-tests were used for data satisfying normality and equal variance assumptions; otherwise, the Mann–Whitney U-test was applied. For the time-course CCK-8 assay, repeated-measures ANOVA with Greenhouse–Geisser correction was employed, followed by Benjamini–Hochberg-adjusted post hoc tests for comparisons between groups at individual time points.

## 3. Results

### 3.1. Prevalence and Impact of Chromosome 7 Gain in Glioma

**Figure 1:**
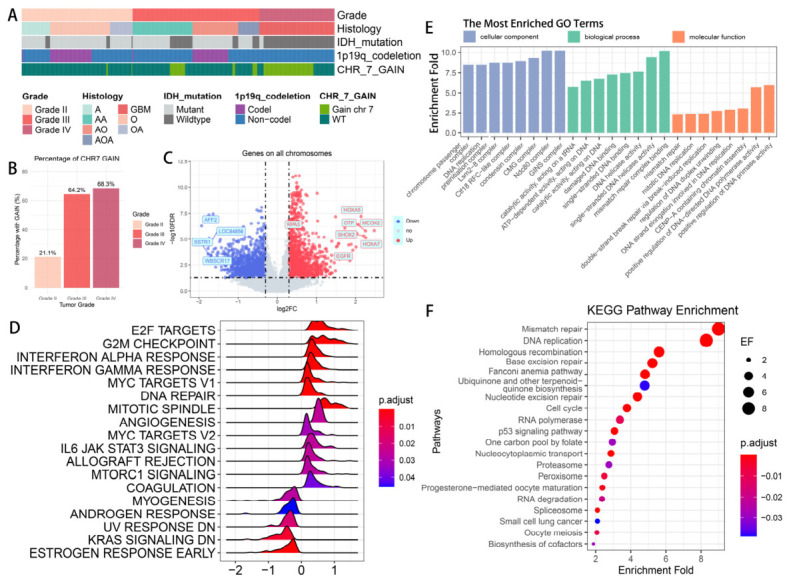
Chromosome 7 gain is enriched in IDH-wildtype glioma and associates with proliferative and DNA-repair programs. (**A**) Cohort overview annotated by WHO grade, histology, IDH status, 1p/19q codeletion, and chr7 gain; chr7 gain predominantly occurs in IDH-wt tumors. (**B**) Proportion of chr7-gain cases by grade in IDH-wt gliomas, showing a step-up from grade II (21.1%) to grade III (64.2%) and grade IV (68.3%). (**C**) Volcano plot of DEGs (DESeq2, BH-adjusted FDR) comparing IDH-wt gliomas with chr7 gain versus chr7 wild type. (**D**) GSEA enrichment of DEGs using hallmark gene sets. (**E**) GO enrichment of DEGs, focusing on cellular component, biological process, and molecular function. (**F**) KEGG pathway analysis of DEGs. Abbreviations: IDH-wt, IDH-wildtype; DEGs, differentially expressed genes.

As shown in Figure 1A, in the TCGA glioma cohort, chr7 gain occurred mainly in IDH-wt tumors, whereas it was rare in IDH-mutant tumors. Across histological subtypes, chr7 gain was enriched in tumors classified as GBM and anaplastic glioma under the 2016 WHO scheme, with the highest proportion seen in GBM. These patterns support focusing subsequent analyses on IDH-wt gliomas to reduce baseline heterogeneity. Within IDH-wt gliomas, chr7 gain was frequent in higher grades: its prevalence exceeded 60% in grade III and grade IV tumors, but was much lower in grade II tumors (21.1%, Figure 1B), suggesting that chr7 gain was associated with malignant grade. Differential expression analysis comparing chr7 gain versus chr7 wildtype IDH-wt tumors revealed broad transcriptomic differences (Figure 1C). Among the most upregulated genes were *EGFR, HOXA5, HOXA7, MEOX2, SHOX2*, and *OTP*, many of which mapped to chromosome 7. GSEA based on these DEGs showed enrichment of hallmark gene sets related to proliferation and inflammatory signaling (Figure 1D). GO enrichment analysis similarly highlighted terms related to chromosome organization, mitotic cell cycle, DNA replication, and DNA break repair (Figure 1E). The top KEGG enriched pathways were again concentrated in multiple DNA repair routes and proliferation-related pathways (Figure 1F). Collectively, these results indicate that chr7 gain is associated with a transcriptional program characterized by cell-cycle progression, proliferative activity, and DNA repair-related features in glioma.

### 3.2. Discovery of Candidate Gene on Chromosome 7

**Figure 2:**
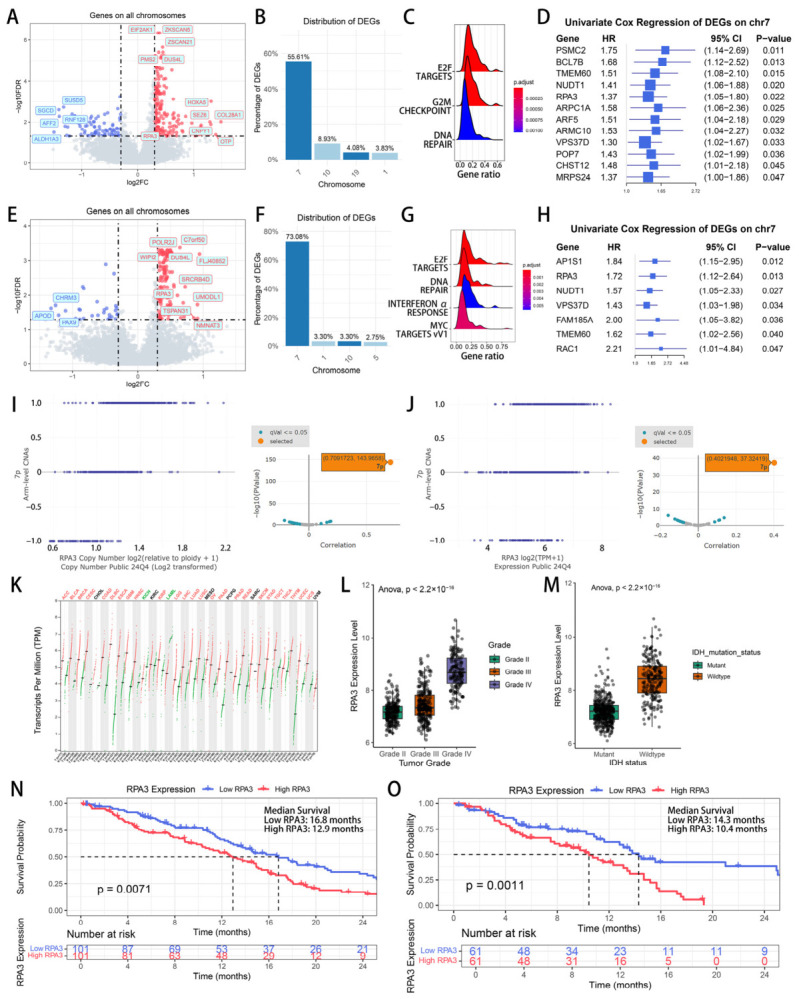
*RPA3* is amplified and overexpressed with chr7 gain and predicts poor prognosis in glioma. (**A**) Volcano plot of DEGs comparing GBMs with chr7 gain versus chr7 wild type (DESeq2, BH-FDR). (**B**) Chromosomal distribution of DEGs. (**C**) GSEA enrichment of DEGs using hallmark gene sets reveals positive enrichment of E2F targets, G2/M checkpoint, and DNA-repair in chr7-gain GBMs. (**D**) Univariate Cox regression of chr7-upregulated candidates identifies prognostic genes; (**E**) Volcano plot of DEGs comparing histologic IDH-wt, Grade 4 GBMs with chr7 gain versus chr7 wild type. (**F**) Chromosomal distribution of DEGs in histologic IDH-wt, Grade 4 GBM. (**G**) GSEA enrichment of DEGs in histologic IDH-wt, Grade 4 GBM. (**H**) Univariate Cox regression of chr7 DEGs in histologic IDH-wt, Grade 4 GBM identifies prognostic genes associated with worse overall survival. (**I**) Scatter plot showing strong concordance between *RPA3* copy-number and arm-level gains of 7p; insets indicate correlation coefficients and significance (r = 0.71; q-value < 0.05). (**J**) Scatter plot showing strong concordance between *RPA3* expression and arm-level gains of 7p; insets indicate correlation coefficients and significance (r = 0.40; q-value < 0.05). (**K**) Pan-cancer comparison using data from TCGA and GTEx shows widespread elevation of *RPA3* in tumors versus matched normal tissues. (**L**) In glioma, *RPA3* expression increases with WHO grade. (**M**) *RPA3* expression is higher in IDH-wt than IDH-mt gliomas. (**N**) Kaplan–Meier analysis shows worse overall survival for patients with high *RPA3* expression (log-rank *p* = 0.0071). (**O**) Kaplan–Meier analysis in histologic IDH-wt, Grade 4 GBM shows significantly shorter overall survival in patients with high *RPA3* expression than in those with low *RPA3* expression (log-rank *p* = 0.0011). Abbreviations: GBM, glioblastoma; DEGs, differentially expressed genes; IDH-wt, IDH-wildtype.

To prioritize chr7-associated candidate genes while minimizing confounding from histological and molecular heterogeneity, we re-stratified the cohort according to the 2021 WHO classification. Specifically, we defined a refined GBM cohort consisting of IDH-wt grade IV GBM together with IDH-wt gliomas harboring 7-gain/10-loss, *EGFR* amplification, or *TERT*-promoter mutation. Within this refined GBM group (WHO 2021 GBM cohort), GBMs with chr7 gain still showed many transcriptional differences compared with chr7-wildtype GBMs (Figure 2A). Among the DEGs, 55.61% were on chromosome 7 and 8.93% were on chromosome 10, with the remainder distributed relatively evenly across other chromosomes (Figure 2B). These data indicate that, after balancing baseline characteristics, chr7 gain still induced substantial transcriptomic alterations, with genes on chr7 accounting for more than half of the expression shift. This distribution pattern is consistent with dosage-associated expression changes across 7p/7q rather than with a single-locus effect.

GSEA performed for DEGs showed pathway activation focused on E2F targets, G2/M checkpoint, and DNA repair (Figure 2C), pointing out that even in the highly proliferative context of GBM, the transcriptional changes driven by chr7 gain remained centered on cell-cycle progression and genome maintenance. DEGs located on chr7 were selected and a univariable Cox analysis were performed to identify genes whose higher expression was linked to poorer survival (Figure 2D). To test whether these observations were robust in a more histologically homogeneous setting, we next performed a sensitivity analysis restricted to histologic GBM, defined here as IDH-wildtype GBM with conventional histopathological features of glioblastoma (Figure 2E–H). In this restricted cohort, chr7-gain tumors still showed a clear transcriptional shift (Figure 2E), and 73.08% of the DEGs were located on chromosome 7 (Figure 2F). GSEA again highlighted pathways including E2F targets and DNA repair (Figure 2G). Several DEGs located on chromosome 7 remained significantly associated with worse overall survival (Figure 2H). Comparing the candidate lists from the univariable Cox analyses of the GBM cohort and the histologic GBM cohort, four prognostic genes overlapped: *RPA3*, *NUDT1*, *VPS37D*, and *TMEM60. VPS37D* and *TMEM60* were not pursued further because their known biological functions showed less direct concordance with the pathway alterations associated with chr7 gain. The remaining two candidates, *RPA3* and *NUDT1*, were both retained as plausible genes of interest. We ultimately prioritized *RPA3* for downstream functional inference and experimental validation because it was directly linked to DNA replication and DNA repair.

Using DepMap, a pan-cancer cell line database with multi-omics data, we confirmed that both *RPA3* copy-number and *RPA3* expression positively tracked the 7p copy-number level (Figure 2I, r = 0.71; q-value less than 0.05; Figure 2J, r = 0.40; q-value less than 0.05), supporting a dosage-associated relationship between chr7 gain and *RPA3* upregulation. Pan-cancer analysis of TCGA and GTEx data further showed that *RPA3* expression was higher in multiple tumor types than in matched normal tissues (Figure 2K). In glioma, *RPA3* expression also varied according to tumor grade and IDH status, with higher levels observed in higher-grade tumors and in IDH-wt disease (Figure 2L,M). These findings place elevated *RPA3* expression within more aggressive clinicopathological contexts. Survival analysis in refined GBM cohort showed that patients with high *RPA3* expression had shorter overall survival than those with low expression (Figure 2N; median OS: 16.8 vs. 12.9 months, log-rank *p* = 0.0071). This survival trend was also found in the histologic GBM cohort (Figure 2O; median OS: 14.3 vs. 10.4 months, log-rank *p* = 0.0011).

### 3.3. Possible Functions of RPA3 in GBM

**Figure 3:**
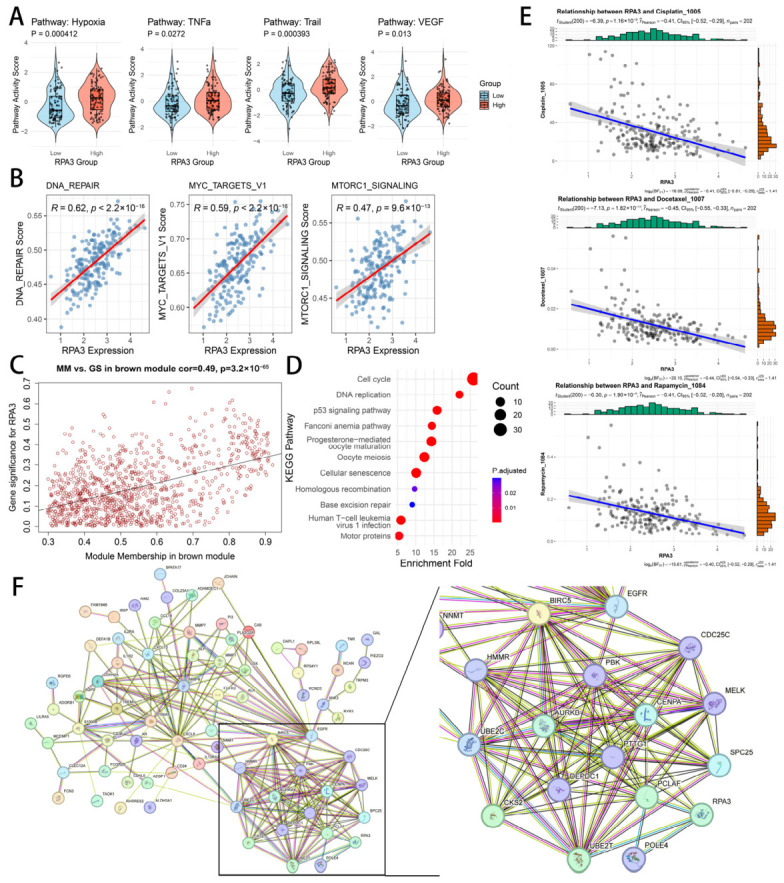
Transcriptomic and network features associated with *RPA3* expression in GBM. (**A**) PROGENy pathway scoring shows higher inferred activity of Hypoxia, TNFα, TRAIL, and VEGF signaling in high-*RPA3* GBMs. (**B**) ssGSEA scoring of hallmark gene sets shows positive correlations between *RPA3* expression and DNA_REPAIR (R = 0.62), MYC_TARGETS_V1 (R = 0.59), and MTORC1_SIGNALING (R = 0.47). (**C**) WGCNA identifies a co-expression module associated with *RPA3*; module membership correlates with gene significance for *RPA3* (cor = 0.49, *p* value < 0.05). (**D**) GSEA enrichment of hub genes in the *RPA3*-correlated module. (**E**) Correlations between *RPA3* expression and predicted drug response (estimated IC50 from public datasets) show that higher *RPA3* is associated with lower predicted IC50 values for cisplatin, docetaxel, and rapamycin. (**F**) STRING-based protein association network constructed from DEGs between *RPA3*-high and *RPA3*-low GBMs identifies a replication- and mitosis-related network centered around *RPA3*. Abbreviations: GBM, glioblastoma; DEGs, differentially expressed genes; PPI, protein–protein interaction.

We further examined the transcriptomic features associated with high *RPA3* expression in the GBM cohort. First, PROGENy pathway scoring was used to infer pathway activity from pathway-responsive genes. Compared with the low-*RPA3* group, tumors with high *RPA3* expression showed higher inferred activity of Hypoxia, VEGF, TNFα, and TRAIL pathways (Figure 3A). Second, ssGSEA was applied to MSigDB hallmark pathways. The pathways most positively correlated with *RPA3* expression were DNA repair (r = 0.62, *p* < 0.001), MYC targets (r = 0.59, *p* < 0.001), and MTORC1 signaling (r = 0.47, *p* < 0.001) (Figure 3B). To evaluate whether these transcriptomic associations were reproducible in an independent dataset, we performed external validation in the CGGA IDH-wt GBM cohort (Supplementary Figure S1). The correlations between *RPA3* expression and ssGSEA pathway scores, as well as the GSEA results of DEGs between the *RPA3*-high and *RPA3*-low groups in CGGA, showed the same overall trends (Supplementary Figure S1).

We next performed WGCNA to identify co-expression modules associated with *RPA3*. *RPA3* expression was used as the trait of interest to identify the most relevant module. As shown in Figure 3C, module membership (MM) was positively correlated with gene significance (GS) for *RPA3* (Figure 3C, r = 0.49, *p* < 0.001), indicating that genes more central to this module also tended to show stronger association with *RPA3* expression. Genes with MM > 0.8 and GS > 0.2 were selected as intramodular hub genes for downstream analysis. GSEA of these genes showed enrichment of cell cycle, DNA replication, p53 signaling, homologous recombination, and base excision repair pathways (Figure 3D). To explore potential associations with drug response, OncoPredict was used to estimate the half-maximal inhibitory concentration (IC50) of multiple agents for each GBM sample. *RPA3* expression showed significant negative correlations with the predicted IC50 values of cisplatin, docetaxel, and rapamycin (Figure 3E).

To further characterize the molecular context associated with *RPA3*, we constructed a STRING-based protein association network using the DEGs between *RPA3*-high and *RPA3*-low GBMs. In this network, *RPA3* was connected with proteins including POLE4, UBE2T, PCLAF, and PBK (Figure 3F). The surrounding network contained multiple proteins related to DNA replication and repair (e.g., PCLAF and POLE4), cell-cycle and mitotic regulation (e.g., AURKB, PBK, MELK, CDC25C, and CKS2), and chromosome segregation (e.g., CENPA, SPC25, HMMR, and PTTG1), and also showed connections with growth- and survival-related factors such as EGFR and BIRC5. These findings place *RPA3* within a transcriptomically associated network enriched for replication- and mitosis-related factors.

### 3.4. RPA3 Is Required for GBM Cell Proliferation and Migration

**Figure 4:**
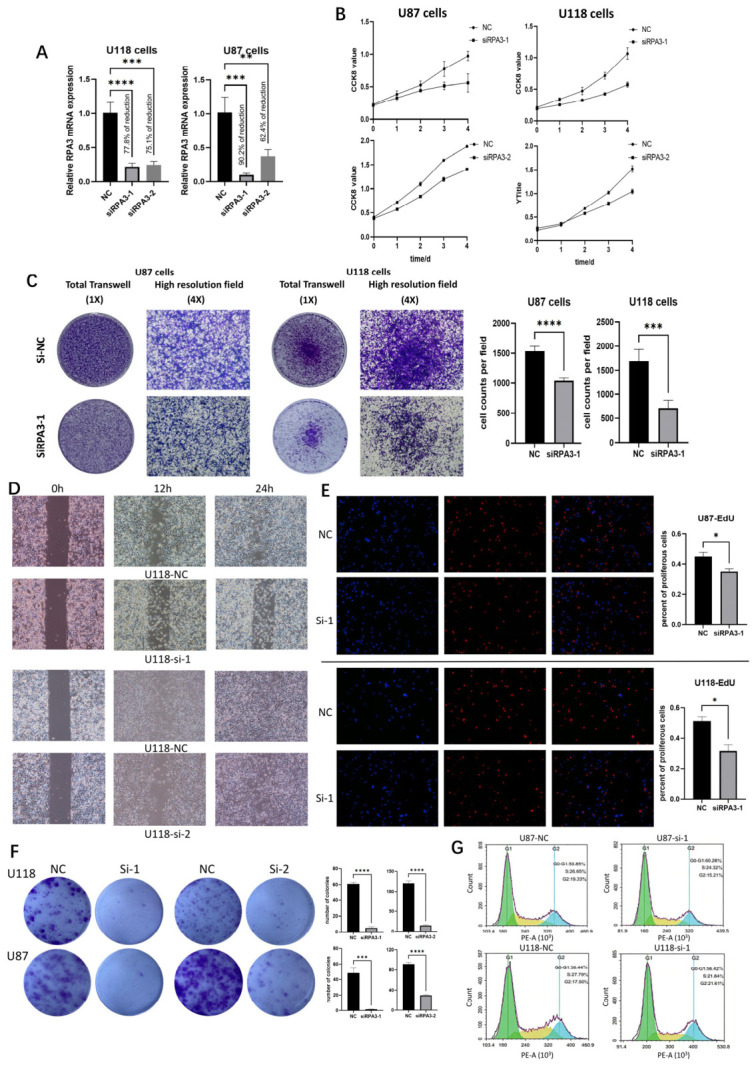
*RPA3* knockdown impairs proliferative and migratory phenotypes in GBM cells in vitro. (**A**) qRT-PCR validation of siRNA-mediated *RPA3* silencing in U118 and U87 cells. (**B**) CCK-8 time-course assay showing reduced cell growth after *RPA3* knockdown. (**C**) Transwell migration assay showing fewer migrated cells in the knockdown groups. (**D**) Wound-healing assay showing delayed gap closure in *RPA3*-silenced U87 and U118 cells. (**E**) EdU incorporation assay showing a reduced proportion of EdU-positive nuclei after *RPA3* knockdown. (**F**) Colony-formation assay showing decreased clonogenic growth after *RPA3* silencing. (**G**) Flow-cytometric cell-cycle analysis showing a decrease in the S-phase fraction after *RPA3* knockdown. Statistical significance is indicated as * *p* < 0.05, ** *p* < 0.01, *** *p* < 0.001, and **** *p* < 0.0001. Abbreviations: GBM, glioblastoma.

To examine the functional relevance of *RPA3* in GBM cells, we silenced *RPA3* with siRNAs in U87 and U118 cells and confirmed efficient knockdown at the mRNA level (Figure 4A). *RPA3* knockdown was associated with reduced cell growth across multiple assays. In the CCK-8 assay, which reflects metabolic activity and short-term viability, the knockdown group showed lower absorbance over time than the control group (Figure 4B). In the EdU assay, *RPA3*-silenced cultures showed a lower fraction of EdU-positive nuclei, consistent with reduced DNA synthesis (Figure 4E). Consistently, flow-cytometric cell-cycle analysis showed a reduced proportion of S-phase cells in the knockdown group (Figure 4G), further supporting an association between *RPA3* depletion and impaired cell-cycle progression. In the colony-formation assay, *RPA3* knockdown led to fewer and smaller colonies than controls, indicating impaired clonogenic growth capacity over time (Figure 4F).

*RPA3* knockdown was also associated with reduced migratory ability. In the transwell migration assay, fewer cells migrated through the membrane in the knockdown group than in the control group (Figure 4C). In the wound-healing assay, *RPA3*-silenced cells showed slower gap closure over the same time interval, leaving a larger residual wound area (Figure 4D).

### 3.5. Single-Cell Analysis Suggests a Preferential Functional Dependence on RPA3 in chr7-Gain Tumor Cells

**Figure 5:**
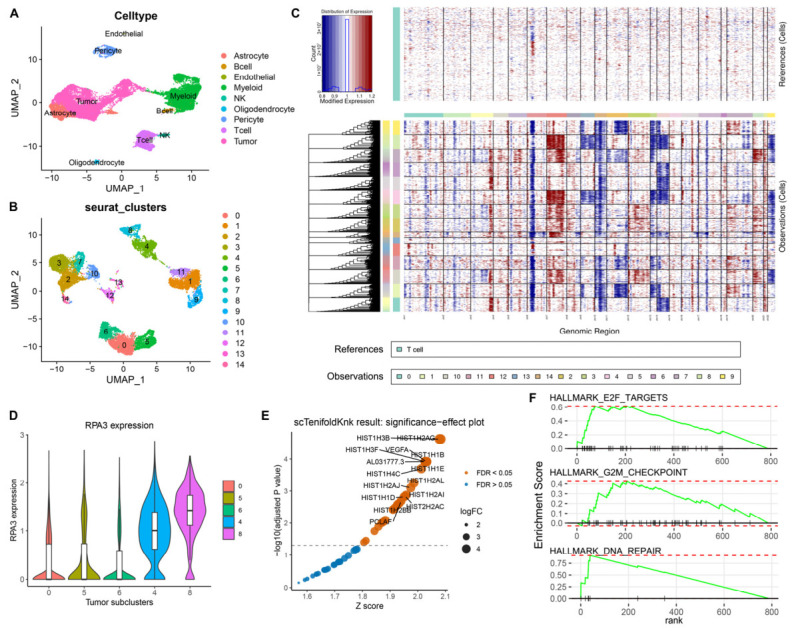
Single-cell analysis suggests preferential functional dependence on *RPA3* in chr7-gain tumor cells. (**A**) UMAP visualization of 27,347 cells, annotated into major cell types. (**B**) UMAP of re-clustered tumor cells, showing refined malignant subclusters. (**C**) InferCNV analysis of the tumor subclusters using T cells as reference cells identifies subclusters 4 and 8 as chr7-gain populations, whereas subclusters 0, 5, and 6 show a chr7-normal-copy pattern. (**D**) Violin plots showing *RPA3* expression across the five selected tumor subclusters. (**E**) scTenifoldKnk significance-effect plot showing the genes most significantly perturbed by in silico *RPA3* knockout in chr7-gain tumor cells; dot color indicates statistical significance (orange, FDR < 0.05; blue, FDR > 0.05), and dot size represents effect size. (**F**) GSEA of the perturbed gene network after virtual *RPA3* knockout in chr7-gain tumor cells. Abbreviations: GBM, glioblastoma.

To further determine whether the functional importance of *RPA3* differs according to chr7 copy-number state, we analyzed a single-cell RNA-sequence dataset comprising 27,347 cells. UMAP projection and cell-type annotation identified the major cellular populations (Figure 5A). We then extracted the tumor cells and performed a second-round clustering to obtain more refined malignant subclusters (Figure 5B). InferCNV analysis revealed clear copy-number heterogeneity among these tumor subclusters: subclusters 4 and 8 showed evident chr7 gain, whereas subclusters 0, 5, and 6 retained a chr7-normal-copy pattern (Figure 5C). Notably, the two groups showed relatively distinct distributions in the UMAP space, indicating greater within-group similarity and supporting transcriptional divergence between chr7-gain and chr7-normal-copy tumor cells.

We next examined *RPA3* expression across these tumor subclusters. *RPA3* expression was markedly higher in the chr7-gain subclusters (Figure 5D), consistent with the dosage-associated expression pattern observed in the bulk data. To further evaluate whether *RPA3* exerts different functional effects in these two cellular contexts, we performed an in silico *RPA3* knockout. Strikingly, significant perturbation of the gene network was detected only in chr7-gain tumor cells, whereas the perturbation in chr7-normal-copy tumor cells was not significant (Supplementary Tables S1 and S2). Among the most affected genes in chr7-gain cells were multiple histone-related genes, together with *VEGFA* and *PCLAF* (Figure 5E). GSEA of the perturbed network further showed that the affected genes were mainly enriched in cell-cycle- and DNA-repair-related pathways (Figure 5F). Collectively, these single-cell results provide supportive evidence that *RPA3* may have greater transcriptomic and network-level relevance in chr7-gain tumor cells than in chr7-normal-copy tumor cells.

## 4. Discussion

This study demonstrates that chr7 gain, as a large-scale amplification, apart from the contributions of well-characterized loci such as *EGFR* and *MET*, is associated with broad transcriptomic alterations and pathway-level changes in GBM. Through transcriptomic profiling of chr7-gain GBMs and correlation with clinical outcomes, we identified a subset of genes on chromosome 7 whose elevated expression is associated with poor patient survival. Among these, *RPA3* was selected for further functional investigation. Our results suggest that *RPA3* is associated with DNA replication- and repair-related programs, while single-cell analysis further indicated that *RPA3* may have greater functional relevance in chr7-gain tumor cells than in chr7-normal-copy tumor cells. Consistently, cellular assays showed that *RPA3* knockdown impaired proliferative and migratory phenotypes in U87 and U118 cells.

When investigating the impact of chr7 gain, after restricting baseline heterogeneity and investigating in the GBM and histologic GBM cohorts, we observed fewer DEGs (Figure 1C vs. Figure 2A,E) and fewer enriched pathways, while the dominant signals remained focused on cell mitosis and DNA repair. This suggests that, in the heterogeneous glioma cohort, part of the DEG signal is confounded by grade-related differences, whereas in the more homogeneous GBM-restricted analyses, the transcriptional changes more likely capture intrinsic chr7-gain-associated features. These results are consistent with an association between chr7 gain and transcriptional programs related to cell-cycle progression and DNA repair. Consistently, previous work showed that in IDH-wt GBM, canonical alterations including chr7 gain were associated with increased abundance of a glial progenitor cell-like malignant state enriched for cycling cells [1]. In addition, a chr7 copy-number-gain study identified *FAM131B-AS2* as a chr7-associated driver of replication-stress tolerance that stabilizes RPA, activates ATR signaling, and protects single-stranded DNA from breakage [37], aligning with our observation that chr7 gain was linked to a more proliferative cellular phenotype.

When choosing target gene between *RPA3* and *NUDT1*, our prioritization of *RPA3* should not be interpreted as implying that *NUDT1* is unimportant. *NUDT1* (also known as MTH1) is itself a biologically credible chromosome 7 candidate in glioma. Prior studies have shown that *NUDT1* is upregulated in GBM, and that its inhibition perturbs mitochondrial homeostasis and enhances oxidative stress [38]. The potential role of *NUDT1* in chr7-gain cells remains worthy of investigation in future studies. Rather, we focused on *RPA3* because it is one of the major subunits of RPA, the major eukaryotic single-stranded DNA-binding complex required for DNA replication, recombination, and repair [13,14], and this molecular function is more directly aligned with the dominant programs emerging from our chr7-gain analyses. Moreover, research showed that the *RPA3* subunit became critically necessary under genotoxic stress. It functioned as a key regulator that tailored RPA complex for S-phase DNA damage tolerance and enabled the RPA complex to effectively manage replication stress and maintain genome stability [39]. Given that GBM cells experience chemoradiotherapy-induced replication stress and increased DNA-lesion burdens, they are likely to exhibit an elevated dependency on *RPA3*.

Building on the established biology of *RPA3* and the RPA complex, our bulk and single-cell transcriptomic analyses allowed us to infer a plausible functional role for *RPA3* in chr7-gain GBM cells. Bulk transcriptomic analyses suggested that high *RPA3* expression is associated with a proliferative and DNA repair-related transcriptional state. Consistently, our in vitro experiments showed that *RPA3* knockdown reduced the fraction of cells entering S phase, supporting a role for *RPA3* in sustaining active cell-cycle progression. Single-cell analysis further supported the relevance of *RPA3* in chr7-gain tumor cells by showing that in silico *RPA3* knockout significantly perturbed gene networks only in chr7-gain tumor cells, with enrichment again centered on E2F, G2/M, and DNA-repair-related programs. Notably, the prominent perturbation of histone-related genes may reflect disruption of replication-coupled chromatin assembly and chromatin restoration after DNA damage, rather than direct effects on canonical DNA-repair enzymes themselves. Collectively, these findings suggest that *RPA3* may be linked to transcriptional programs related to replication stress adaptation in chr7-gain GBM; however, this interpretation remains inferential and requires direct experimental assays to confirm its specific functions in future studies.

The predicted lower IC50 values for cisplatin, docetaxel, and rapamycin in *RPA3*-high tumors should, however, be interpreted cautiously, because these estimates were transcriptome-based rather than directly validated experimentally. In our study, *RPA3* knockdown reduced EdU incorporation and was accompanied by a lower S-phase fraction, supporting the idea that *RPA3* contributes to S-phase progression and ongoing DNA synthesis in GBM cells. One possible interpretation is that high *RPA3* expression is associated with transcriptional features consistent with replication-stress adaptation, consistent with evidence that cancer cells can enhance fork stabilization and checkpoint responses to survive replication-stress-inducing conditions [19]. At the same time, this state may also reflect greater dependence on the replication-stress response itself. A key function of ATR is to prevent global exhaustion of RPA; when replication stress intensifies, excessive ssDNA generation can deplete available RPA, leading to widespread fork breakage and replication catastrophe [40]. The replication checkpoint protects stressed cells by preventing exhaustion of limiting replication factors [41]. In this context, the association between high *RPA3* and lower predicted cisplatin IC50 may reflect a high-stress equilibrium: although these cells may possess stronger stress-buffering capacity at baseline, they may also operate closer to the upper limit of replication-stress tolerance.

Prior studies have already implicated *RPA3* in several malignancies, but the nature of that evidence has been heterogeneous. In gastric cancer and bladder urothelial carcinoma, elevated *RPA3* expression was mainly linked to adverse clinicopathological features and poorer survival [20,21]. In nasopharyngeal carcinoma, *RPA3* was associated with radioresistance [22], whereas in hepatocellular carcinoma and lung adenocarcinoma, experimental studies further connected *RPA3* to DNA repair- or treatment-response-related phenotypes [42,43]. A glioma study reported that *RPA3* is overexpressed and promotes proliferation, migration, and invasion, partly through PI3K-AKT-mTOR signaling [23]. These studies support an oncogenic or therapy-relevant role for *RPA3* across multiple cancer types. Our study extends this literature by examining *RPA3* in the context of broad chromosome 7 gain in IDH-wt glioma. We identified *RPA3* as a dosage-sensitive chromosome 7 candidate associated with proliferation- and DNA repair-related programs, and raised the possibility that increased *RPA3* expression may contribute to the selective advantage associated with chr7 gain.

Several limitations should be acknowledged. First, although the single-cell analysis provides preliminary support that *RPA3* may be more functionally relevant in chr7-gain tumor cells, this finding remains inferential and requires further validation in patient-derived GBM models or engineered systems with diverse and well-defined chr7 copy-number states. Second, the proposed role of *RPA3* in chr7-gain GBM cells is mainly based on transcriptomic, co-expression, and STRING-based protein functional network analyses. Although these analyses consistently link *RPA3* to DNA replication, cell-cycle progression, and DNA repair-related programs, direct experimental assays will be needed to confirm its specific functions in DNA replication and repair in chr7-gain GBM cells.

Integrating copy-number, expression, and outcome data, our study identifies *RPA3* as a chromosome 7-encoded, dosage-sensitive locus associated with cell-cycle progression and DNA-repair-related programs in GBM. These findings support the possibility that chr7 gain may confer a selective advantage, at least in part, through increased *RPA3* expression that helps sustain rapid cycling under genotoxic stress. *RPA3* may, therefore, serve as a candidate biomarker of aggressiveness and as a potential therapeutic target for chr7-gain GBM that warrants further investigation.

## Data Availability

The datasets analyzed during the current study are publicly available from The Cancer Genome Atlas (TCGA) via the NCI Genomic Data Commons (GDC) Data Portal (https://portal.gdc.cancer.gov/) and can be accessed using the GDC data transfer tool or the GDC Application Programming Interface.

## Supplementary Materials

The following supporting information can be downloaded at: https://www.mdpi.com/article/10.3390/biomedicines14051014/s1, Figure S1. External validation of *RPA3*-associated transcriptomic features in the CGGA IDH-wildtype GBM cohort. (A) ssGSEA analysis showed significant positive correlations between *RPA3* expression and pathway scores for DNA repair, MYC targets V2, MYC targets V1, and E2F targets. Spearman correlation coefficients and FDR-adjusted p values are shown in each panel. (B) GSEA enrichment comparing *RPA3*-high and *RPA3*-low tumors. Table S1. Gene regulatory network perturbation results after in silico *RPA3* knockout in chr7-gain tumor cells. This table summarizes the scTenifoldKnk-derived perturbation results for genes affected by virtual *RPA3* knockout in chr7-gain tumor subclusters. Genes are listed with corresponding perturbation metrics and statistical significance values. Table S2. Gene regulatory network perturbation results after in silico *RPA3* knockout in chr7-normal-copy tumor cells. This table summarizes the scTenifoldKnk-derived perturbation results for genes affected by virtual *RPA3* knockout in chr7-normal-copy tumor subclusters. Genes are listed with corresponding perturbation metrics and statistical significance values.

## Abbreviations

AKT, protein kinase B; ANOVA, analysis of variance; BH, Benjamini–Hochberg; CCK-8, Cell Counting Kit-8; CI, confidence interval; CNS, central nervous system; cDNA, complementary DNA; CNA, copy-number alteration; DEGs, differentially expressed genes; DMEM, Dulbecco’s Modified Eagle Medium; *EGFR*, epidermal growth factor receptor; FBS, fetal bovine serum; GBM, glioblastoma; GDC, Genomic Data Commons; GDSC2, Genomics of Drug Sensitivity in Cancer (version 2); GEPIA2, Gene Expression Profiling Interactive Analysis 2; GISTIC2.0, Genomic Identification of Significant Targets in Cancer 2.0; GO, Gene Ontology; GSVA, Gene Set Variation Analysis; GTEx, Genotype-Tissue Expression; GSEA, Gene Set Enrichment Analysis; HR, hazard ratio; IC50, half-maximal inhibitory concentration; IDH, isocitrate dehydrogenase; IDH-mt, IDH-mutant; IDH-wt, IDH-wildtype; KEGG, Kyoto Encyclopedia of Genes and Genomes; KM, Kaplan–Meier; *MET*, *MET* proto-oncogene, receptor tyrosine kinase; MM, module membership; mOS, median overall survival; MSigDB, Molecular Signatures Database; mTOR, mechanistic target of rapamycin; MTORC1, mechanistic target of rapamycin complex 1; MYC, MYC proto-oncogene; NC, negative control; NCI, National Cancer Institute; OS, overall survival; PBS, phosphate-buffered saline; PDGFA, platelet-derived growth factor subunit A; PI3K, phosphoinositide 3-kinase; PPI, protein–protein interaction; PROGENy, Pathway RespOnsive GENes; qRT-PCR, quantitative reverse transcription polymerase chain reaction; RPA, replication protein A; *RPA3*, replication protein A3; siRNA, small interfering RNA; ssDNA, single-stranded DNA; ssGSEA, single-sample Gene Set Enrichment Analysis; STRING, Search Tool for the Retrieval of Interacting Genes/Proteins; TCGA, The Cancer Genome Atlas; TERT, telomerase reverse transcriptase; TNFα, tumor necrosis factor alpha; TRAIL, TNF-related apoptosis-inducing ligand; TPM, transcripts per million; VEGF, vascular endothelial growth factor; WGCNA, weighted gene co-expression network analysis; WHO, World Health Organization; chr7, chromosome 7; chr7 gain, chromosome 7 gain; 10 loss, chromosome 10 loss.
